# Mitochondrial Unfolded Protein Response Gene *Clpp* Is Required for Oocyte Function and Female Fertility

**DOI:** 10.3390/ijms25031866

**Published:** 2024-02-03

**Authors:** Yagmur Ergun, Aysegul Gizem Imamoglu, Mauro Cozzolino, Cem Demirkiran, Murat Basar, Akanksha Garg, Raziye Melike Yildirim, Emre Seli

**Affiliations:** 1 Department of Obstetrics, Gynecology, and Reproductive Sciences, Yale School of Medicine, New Haven, CT 06510, USA; 2 IVIRMA Global Research Alliance, IVIRMA New Jersey, Marlton, NJ 07920, USA; 3 IVIRMA Global Research Alliance, IVI Roma, 00169 Rome, Italy; 4 IVIRMA Global Research Alliance, IVI Foundation, Instituto de Investigación Sanitaria La Fe (IIS La Fe), 46026 Valencia, Spain; 5 Department of Obstetrics, Gynecology, and Reproductive Sciences, Yale Fertility Center, Orange, CT 06477, USA; 6 Department of Metabolism, Digestion and Reproduction, Imperial College London, London SW7 2BX, UK; 7 IVIRMA Global Research Alliance, IVIRMA New Jersey, Basking Ridge, NJ 07920, USA

**Keywords:** mitochondrial stress response, mitochondrial dysfunction, female fertility, oocyte function, CLPP

## Abstract

Mitochondrial unfolded protein stress response (mtUPR) plays a critical role in regulating cellular and metabolic stress response and helps maintain protein homeostasis. Caseinolytic peptidase P (CLPP) is one of the key regulators of mtUPR and promotes unfolded protein degradation. Previous studies demonstrated that global deletion of *Clpp* resulted in female infertility, whereas no impairment was found in the mouse model with targeted deletion of *Clpp* in cumulus/granulosa cells. These results suggest the need to delineate the function of *Clpp* in oocytes. In this study, we aimed to further explore the role of mtUPR in female reproductive competence and senescence using a mouse model. Oocyte-specific targeted deletion of *Clpp* in mice resulted in female subfertility associated with metabolic and functional abnormalities in oocytes, thus highlighting the importance of CLPP-mediated protein homeostasis in oocyte competence and reproductive function.

## 1. Introduction

Through the past few decades, the significant role of mitochondria in male and female reproductive function has been demonstrated in both animal and human models. Emerging evidence suggests that pre-implantation embryos’ developmental competence is in part determined by mitochondrial biogenesis and bioenergetics [1,2]. The number of mitochondria and the amount of mitochondrial DNA (mtDNA) are biologically significant for mammalian oocyte competency and early embryo development [3]. Dysfunctional mitochondria can subsequently impact physiological reproductive processes, resulting in defective oocyte maturation, impaired fertilization [4], and altered embryonic developmental competence [5,6].

Mitochondrial unfolded protein response (mtUPR) is a transcriptional response that is activated in response to the accumulation of misfolded proteins and mitochondrial stress. Metabolic insults, such as reactive oxygen species (ROS), may generate misfolded proteins, which lead to the activation of the mtUPR pathway. In turn, mtUPR promotes a transcriptional response to increase protein folding and suppress the synthesis of new proteins until homeostasis is achieved. This pathway was first characterized in *Caenorhabditis elegans* [7], where caseinolytic peptidase P (CLPP) was found to cleave unfolded or misfolded proteins in the mitochondria and export them to cytosol [8,9]. These cleaved proteins activate stress-activated transcription factor 1 (ATFS1), which subsequently regulates the formation of defective proventriculus homolog protein (DVE1) and Ubiquitin-like 5 (UBL5) complexes [7,10]. Furthermore, activated ATSF1 induces the transcription of mitochondrial chaperones, such as heat shock protein 6 (HSP6) and heat shock protein 10 (HSP10) [9].

CLPP is the key regulator of the mtUPR pathway. Mutations in the mitochondrial protease CLPP result in Perrault syndrome type 3 (PRLTS3), an autosomal recessive disorder characterized by complete infertility due to female primary ovarian insufficiency and male azoospermia after meiotic arrest at diplotene, with sensorineural deafness, ataxia, leukodystrophy, and neuropathy [11,12,13]. Previous studies have shown that global germline *Clpp* deficiency in female mice results in impaired oocyte maturation, abnormal formation of two-cell embryos, and blastocyst development failure, ultimately leading to infertility [14,15]. Although these data allude to the relevance and importance of *Clpp* in maintaining reproductive function, it remains unclear whether it is the gonadal or somatic cells that are predominantly affected by the lack of *Clpp* and subsequent downstream effects on the mtUPR pathway. To further characterize this, Esencan et al. investigated the role of CLPP in granulosa/cumulus cells and found that *Clpp* deletion specifically in these cells did not affect female fertility through tissue-specific mtUPR [16]. This raises the question regarding exactly where, and how, *Clpp* deletion contributes to the abnormal reproductive milieu seen in global knockout mice models [14,15].

In this study, we aimed to investigate whether the targeted deletion of *Clpp* in mouse oocytes caused impaired reproductive competence of females through dysfunctional mitochondrial stress response. Our findings demonstrate that a lack of *Clpp* causes metabolic and functional abnormalities in oocytes and results in decreased female fertility.

## 2. Results

### 2.1. Oocyte-Specific Targeted Deletion of Clpp Results in Female Subfertility

Mice with oocyte-specific deletion of *Clpp* were generated by mating *Clpp*^flox/flox^ female mice with *Clpp*^flox^/Zp3-Cre male mice (Supplementary Figure S1). Sexually mature *Clpp^−/−^* and WT female mice (n = 5 for 2-month-old, and n = 4 for 6-month-old) were subjected to a continuous mating study with WT males of proven fertility (female/male; 1:1) for 12 weeks.

After 12 weeks of mating, 2-month-old *Clpp^−/−^* mice were found to be subfertile when compared to WT females (7.2 ± 1.98 vs. 14.40± 1.66 pups per female; *p* < 0.05) (Table 1). When we assessed the fertility of older of (6-month-old) *Clpp^−/−^* mice, we found that they had a more pronounced subfertility and a decreased number of litters per female and pups per female in comparison to WT (1.25 ± 1.19 vs. 3 ± 0 litter per female and 6 ± 2.44 vs. 17.25 ± 3.49 pups per female; *p* < 0.05) (Table 1).

### 2.2. Lack of Clpp in Oocytes Alters Follicle Development

**Figure 1:**
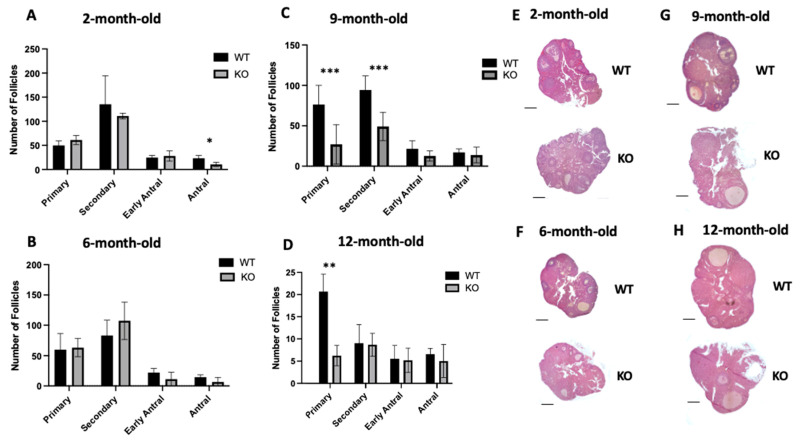
Assessment of follicle development in mice with targeted deletion of *Clpp* in oocytes. Follicle counts in (**A**) 2-month-old, (**B**) 6-month-old, (**C**) 9-month-old, and (**D**) 12-month-old *Clpp*^−/−^ and WT mice ovaries (n = 3 for each genotype and time point). Data presented as mean ± SEM. Representative micrographs of (**E**) 2-month-old, (**F**) 6-month-old, (**G**) 9-month-old, and (**H**) 12-month-old *Clpp*^−/−^ and WT mice ovarian sections stained with hematoxylin and eosin. * *p* < 0.05,** *p* < 0.001, *** *p* < 0.001. Scale bars: 100 um.

Follicle development was assessed in 2-, 6-, 9-, and 12-month-old *Clpp^−/−^* and WT female mice (Figure 1). In 2-month-old mice, *Clpp^−/−^* females showed a similar number of primordial, primary, secondary, and early antral follicles compared to WT mice (29.00 ± 4.35 vs. 31.33 ± 6.12 (*p* = 0.77); 61.20 ± 5.41 vs. 49.83 ± 5.60 (*p* = 0.21); 104.2 ± 8.09 vs. 135.5 ± 3.44 (*p* = 0.54); and 28.00 ± 8.66 vs. 24.67 ± 6.11 (*p* = 0.65) (Figure 1A,E). However, the antral follicles were significantly decreased (10.50 ± 2.51 vs. 23.17 ± 2.51 *p* < 0.05).

When we assessed the follicle development in 6-month-old mice, all follicle numbers were similar compared to WT (33.33 ± 2.80 vs. 33.83 ± 0.44 (*p* = 0.87); 63.23 ± 8.55 vs. 59.83 ± 5.33 (*p* = 0.85); 107.3 ± 7.89 vs. 83.00 ± 4.64 (*p* = 0.1); 11.17 ± 6.69 vs. 22.00 ± 3.96 (*p* = 0.25); and 6.50 ± 4.36 vs. 14.50 ± 2.25 (*p* = 0.20) for primordial, primary, secondary, early antral, and antral, respectively) (Figure 1B,F).

In 9-month-old KO mice, we found the number of primordial, early antral, and antral follicles were similar compared to WT (11.00 ± 4.65 vs. 27.33 ± 1.33 (*p* = 0.18); 12.67 ± 3.65 vs. 21.50 ± 5.79 (*p* = 0.46); and 13.83 ± 5.60 vs. 17.00 ± 2.56 (*p* = 0.79), respectively), while primary and secondary follicles were significantly decreased (27.00 ± 4.07 vs. 76.33 ± 3.71 (*p* < 0.001); 49.17± 9.10 vs. 94.33 ± 9.09 (*p* < 0.001), respectively) (Figure 1C,G).

In 12-month-old mice, we did not detect a difference in the number of follicles at any of the developmental stages, except for a significant decrease in primary follicles in *Clpp^−/−^* ovaries compared to WT. The number of follicles was 9 ± 2.25 vs. 6.16 ± 1.01 (*p* = 0.31); 6 ± 1.25 vs. 19.83 ± 2.16 (*p* = 0.005); 8.33 ± 1.42 vs. 8.66 ± 2.33 (*p* = 0.90); 5 ± 1.52 vs. 5.33 ± 1.64 (*p* = 0.88); and 4.83 ± 2.04 vs. 6.33 ± 0.72 (*p* = 0.52) for primordial, primary, secondary, early antral and antral, respectively (Figure 1D,H).

### 2.3. Oocyte-Specific Targeted Deletion of Clpp Results in Altered Oocyte Development

**Figure 2:**
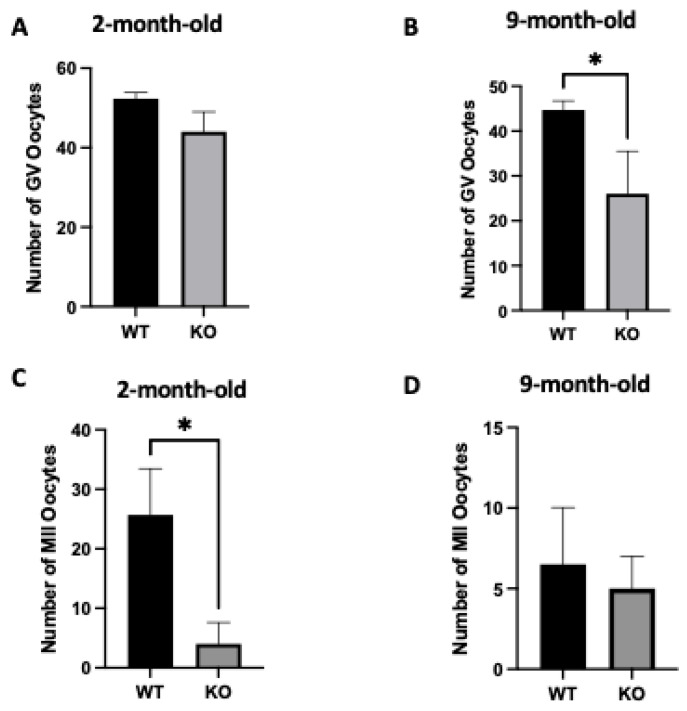
Assessment of oocyte development in oocyte-specific *Clpp^−/−^* mice. Number of GV oocytes in (**A**) 2-month-old and (**B**) 9-month-old *Clpp*^−/−^ and WT mice. Number of MII oocytes in (**C**) 2-month-old and (**D**) 9-month-old *Clpp*^−/−^ mice. KO: *Clpp*^−/−^; * *p* < 0.05.

The production of germinal vesicle stage (GV, prophase I) and mature (MII) oocytes in 2-month-old and 9-month-old mice was evaluated after injection with 10 IU of PMSG or 10 IU of PMSG and hCG. In 2-month-old *Clpp^−/−^* female mice, GV numbers were different, but the difference was not statistically significant compared to WT (44 ± 2.8 vs. 52.3 ± 0.8; *p* = 0.05) (Figure 1A). However, 9-month-old *Clpp^−/−^* mice demonstrated a significant decrease in the number of GV oocytes compared to WT (26 ± 5.5 vs. 44 ± 1.2; *p* < 0.05) (Figure 2B).

The number of MII oocytes was significantly lower in 2-month-old *Clpp^−/−^* mice compared with WT (4 ± 1.15 vs. 25.67 ± 4.48; *p* < 0.05), whereas the difference was not significant at 9 months (5 ± 1.15 vs. 6.5 ± 2.5; *p* = 0.57) (Figure 2C,D).

### 2.4. Clpp Depletion in Oocytes Results in Mitochondrial Dysfunction

**Figure 3:**
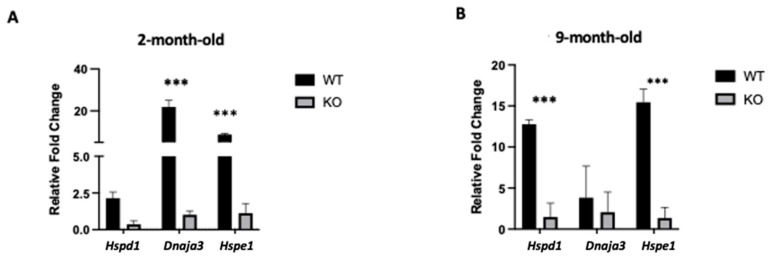
Unfolded protein response in *Clpp*^−/−^ oocytes is impaired. Expression levels of mitochondrial unfolded protein response (mtUPR) genes were assessed using qRT-PCR in GV oocytes collected from (**A**) 2-month-old and (**B**) 9-month-old *Clpp*^−/−^ and WT female mice. *Hspd1*: Heat Shock Protein Family D (Hsp60) Member 1, *Dnaja3*: DnaJ Heat Shock Protein Family (Hsp40) Member A3, *Hspe1*: Heat Shock Protein Family E (Hsp10) Member 1. *** *p* < 0.001.

Mitochondrial unfolded protein response (mtUPR) genes and *Hspd1*, *Hspe1*, and *Dnaja3* expression levels were also evaluated in *Clpp^−/−^* oocytes (Figure 3). In 2-month-old *Clpp^−/−^* mouse oocytes, we found lower expressions of *Hspe1* (1.12 ± 0.37 vs. 8.75 ± 0.29; *p* < 0.001) and *Dnaja3* (1.01 ± 0.15 vs. 21.88 ± 1.83; *p* < 0.001), while *Hspd1* expression was similar to WT (0.3 ± 0.23 vs. 2.13 ± 0.14; *p* = 0.13) (Figure 3A). When we assessed 9-month-old mice, we had similar findings with lower expressions of *Hspd1* (1.47 ± 0.97 vs 12.78 ± 0.30; *p* < 0.001) and *Hspe1* (1.36 ± 0.74 vs. 15.44 ± 0.93; *p* < 0.001) in *Clpp^−/−^* oocytes compared to WT, while *Dnaja3* was unchanged (2.05 ± 1.41 vs. 3.83 ± 2.22; *p* = 0.53) (Figure 3B).

**Figure 4:**
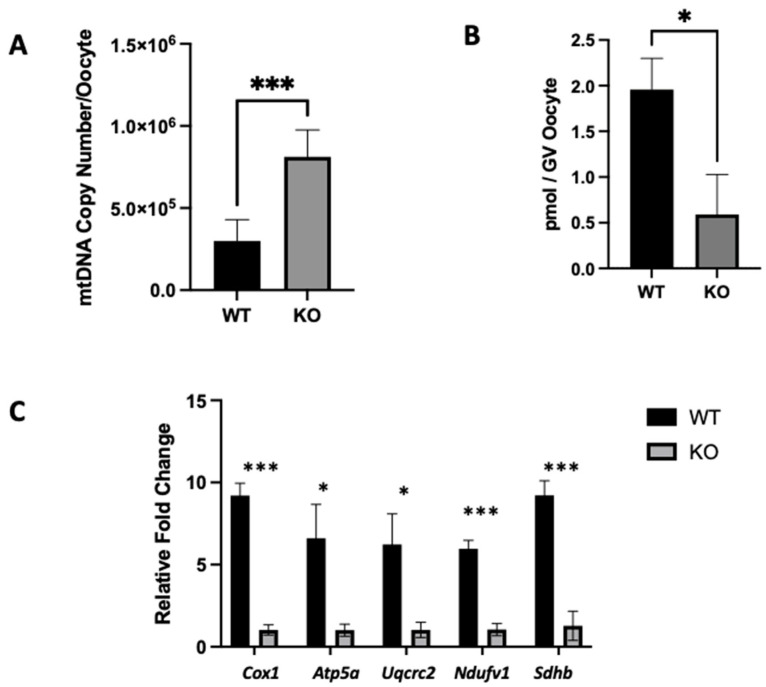
Mitochondrial function and dynamics are impaired in *Clpp*^−/−^ oocytes. (**A**) mtDNA copy number was determined by qPCR in individual GV oocytes from 2-month-old *Clpp*^−/−^ and WT female mice. (**B**) ATP content of individual oocytes was assessed using the ATP bioluminescent cell assay. (**C**) Expression levels of electron transport chain genes were determined using qRT-PCR in GV oocytes from 2-month-old *Clpp*^−/−^ and WT female mice. *Cox1*: cytochrome c oxidase subunit I, *Atp5a1*: Atp synthase F1, subunit alpha, *Uqcrc2*: ubiquinol-cytochrome C reductase core protein 2, *Ndufv1*: NADH: ubiquinone oxidoreductase core subunit V1, *Sdhb*: succinate dehydrogenase complex iron sulfur subunit B, * *p* < 0.05, *** *p* < 0.001.

We also assessed mitochondrial function in *Clpp^−/−^* oocytes compared to WT. In GV oocytes from 2-month-old *Clpp*^−/−^ female mice, the mtDNA copy number was found to be significantly higher when compared to WT (812,499 ± 66.79 vs. 298,950 ± 53.27; *p* < 0.001) (Figure 4A). Significantly decreased ATP production (0.5 ± 0.44 vs. 1.95 ± 1.19; *p* < 0.05) (Figure 4B) was also found in *Clpp*^−/−^ oocytes when compared to WT. When we assessed expression levels of electron transport chain genes (*Cox1*, *Atp5a1*, *Uqcrc2*, *Ndufv1*, and *Sdh*b), we found that *Clpp*^−/−^ oocytes showed decreased expression compared to WT (1.30 ± 0.17 vs. 9.20 ± 0.43 (*p* < 0.001); 1.01 ± 0.21 vs. 6.60 ± 1.18 (*p* < 0.05); 1.30 ± 0.26 vs. 6.23 ± 1.07 (*p* < 0.05); 1.50 ± 0.21 vs. 5.96 ± 0.29 (*p* < 0.001); and 1.28 ± 0.51 vs. 9.21 ± 0.51 (*p* < 0.001), respectively) (Figure 4C).

## 3. Discussion

*Clpp* plays a critical role in mtUPR by regulating the degradation of unfolded and misfolded proteins in mitochondria. Previous studies demonstrated that global deletion of *Clpp* results in female infertility and accelerated depletion of ovarian follicular reserve consistent with a diminished ovarian reserve phenotype [14]. Interestingly, a subsequent study by Esencan et al. demonstrated that the targeted deletion of *Clpp* in granulosa/cumulus cells does not affect female fertility [16], thereby suggesting that the primary role of *Clpp* in enabling reproductive function may lie elsewhere, such as in oocytes. In the current study, we aimed to establish whether the targeted deletion of *Clpp* in oocytes affects female infertility.

We found that the oocyte-specific targeted deletion of *Clpp* causes female mice to be subfertile, generating a significantly lower number of pups after 12 weeks of observation starting at both 2 and 6 months of age (Table 1). Our findings are consistent with a recent study by Li et al. that reported subfertility using a similar conditional knockout strategy [17]. Importantly, the subfertility was more pronounced in 6-month-old *Clpp^−/−^* mice, consistent with an accelerated ovarian aging phenotype, which was also suggested by a prior study with the global germline deletion of *Clpp* (causing deletion in all cells) [14].

The impact of the oocyte-specific targeted deletion of *Clpp* on follicle development was limited compared to what was observed in the prior global germline *Clpp* knockout study [14]. In young (2-month-old) mice, we found a similar number of follicles in most stages, with only a significant decrease in the number of antral follicles (Figure 1A). While this is a much milder phenotype compared to the global germline knockout [14], it is not surprising, as the *ZP3-Cre*-mediated deletion of *Clpp* is activated in the oocyte after the transition from primordial to the primary follicle stage [18,19]. As expected, our findings are quite similar to what was reported in a recent study with the conditional oocyte-specific deletion of *Clpp*, where an increase in atretic follicles was observed compared to the wild type and all stages of follicular development were similar between the groups [17].

Mitochondrial proteolytic enzymes (proteases), which break down misfolded proteins, are crucial in promoting the response to mitochondrial stress [20]. Previous studies have demonstrated that the lack of one such protease, termed mitochondrial ATP-dependent Lon protease 1 (LONP1), can result in the apoptosis of mouse oocytes. This is thought to be due to increased apoptosis-inducing factor mitochondria-associated 1 (AIFM1) translocation from the cytoplasm to the nucleus. Additionally, women with pathogenic variants of LONP1 failed to develop large antral follicles [21]. There is evidence highlighting common substrates shared by LONP1 and CLPP, such as serine hydroxymethyltransferase 2 (SHMT2). Under conditions of metabolic stress, the inhibition of LONP1 and CLPP together results in an increase in the quantity of SHMT2 unfolded protein and improved sensitivity to SHMT2 inhibitors, leading to a considerable reduction in cell growth and an increase in cell death [22]. It is possible that the absence of CLPP in young reproductive age mice leads to the activation of such mechanisms to cause a decrease in antral follicles.

In older reproductive age mice (9 months old and 12 months old), we found primary follicles to be significantly decreased in *Clpp*^−/−^ female mice (Figure 1). Consistent with our findings, it was also reported that global knockout female mice developed a lower number of primordial and primary follicles [14]. As the oocyte begins to grow and differentiate from the primary follicle stage onwards, there is a progressive increase in oocyte RNA synthesis. Some genes, including those encoding the zona pellucida 3 protein (ZP3), begin to be transcribed and translated at the primary follicle stage [23,24]. Therefore, the primary follicle is the stage when targeted deletion of *Clpp* in oocytes took place in our model. The decreased number of primary follicles in 9- and 12-month-old ovaries could be equivalent to the human diminished ovarian reserve phenotype [25].

When we assessed mitochondrial function in *Clpp*-deficient oocytes, we found that the mtDNA copy number was significantly higher (Figure 4A), whereas the ATP production and expression of electron transport chain (ETC) genes were down-regulated (Figure 3C and Figure 4B). Mitochondria exist in a fine equilibrium. Disturbance of the mitochondrial milieu, such as increased production of ROS, can lead to an aggregation of unfolded proteins and mitochondrial dysfunction. Such mitochondrial dysfunction may cause a change in the mtDNA copy number in the cell [14,26,27]. As expected, we found that *Clpp*^−/−^ female mice demonstrated a higher mtDNA copy number, which is consistent with the results of the global knockout mouse model [14]. Furthermore, the oxidative phosphorylation, the primary mechanism through which mitochondria generate ATP [28], was down-regulated, and lower ATP levels were found in *Clpp*^−/−^ female mice oocytes, consistent with mitochondrial dysfunction [29] and impaired reproductive potential [30,31]. In humans, higher ATP content in developing oocytes and embryos is associated with better reproductive outcomes amongst patients diagnosed with infertility [32]. Our findings, in keeping with a recent study that also used conditional deletion of *Clpp* in oocytes [17], demonstrate how *Clpp*^−/−^ female mice possess a certain level of mitochondrial dysfunction, which may contribute to their subfertile profile.

To further characterize mitochondrial function in *Clpp*^−/−^ oocytes, we assessed the expression levels of mitochondrial unfolded protein response genes in 2- and 9-month-old mice (Figure 3). Heat shock proteins (HSPs) play a key role in maintaining homeostasis, and limiting cellular aging processes [33]. Although the definite role of heat shock proteins in embryogenesis is still unknown, studies suggested that blastocyst development is promoted by HSP60 and HSP70, and their inhibition can alter normal development and increase apoptosis-mediated cell death in embryos [34,35]. Our results indicate that the lack of *Clpp* in oocytes could contribute to a dysfunctional response to stress, correlating with the impaired expression of HSP genes seen in both 2- and 9-month-old mice.

Emerging evidence strongly supports the role of metabolic determinants in causing subfertility. Kisspeptins (KPs) control the initiation of puberty, gonadotropin production mediated by sex hormones, and gonadal function maintenance [36]. A wide range of physiological and pathological processes have been linked to KP peripheral activity in female reproductive organs [37]. Furthermore, new research implicates KPs in a wide range of intricate systemic biological processes, including the formation and function of endometrial glands [38], the recruitment and proliferation of follicles [39], and the implantation and placentation of embryos [40]. A recent study by Mattam et al. [39] found that kisspeptin-10 induces autophagy and mitophagy independently of the mammalian target of rapamycin (mTOR) through the signaling pathways of calcium, Ca^2+^/CaM-dependent protein kinase β (CaMKKβ), AMP-activated protein kinase (AMPK), and Unc-51-like autophagy activating kinase (ULK1). Concordantly, probable benefits associated with KPs in preserving mitochondrial health and as a potential treatment for hippocampal-related deficits, such as memory loss, cognitive aging, and other diseases connected to dysfunctional mitochondria, are being investigated.

Another pathway that links metabolic dysfunction to reproductive impairment involves leptin, an adipocyte-derived peptide hormone that regulates energy balance. Leptin affects female reproductive function through its effects on the hypothalamic–pituitary–ovarian axis [41]. In animal models, the targeted deletion of leptin is associated with obesity, type II diabetes, and infertility [42,43]. Similar to the suppression of *Hsp60* expression, we observed in oocytes with the targeted deletion of *Clpp* that HSP60 expression is reduced in obese, diabetic mice due to a lack of proper leptin signaling and can be restored by leptin treatment [44]. Importantly, the reduced expression of the mitochondrial chaperone HSP60 is associated with mitochondrial dysfunction in diabetes and insulin resistance [45].

In this study, we expanded our understanding of the role of *Clpp* and mtUPR in female reproduction using a mouse model with the targeted deletion of *Clpp* in oocytes. The lack of *Clpp* resulted in metabolic and functional abnormalities in oocytes and caused decreased female fertility. Whether this pathway can be exploited as a diagnostic or therapeutic target within human reproduction remains to be further investigated. However, it is becoming evident that *Clpp* functioning is crucial for maintaining normal reproductive function.

## 4. Material and Methods

### 4.1. Animals

All mice care, breeding, and experimental protocols were conducted according to the Yale University Animal Research Requirements, and the used protocols were approved by the Institutional Animal Care and Use Committee (Protocol #2020-11207).

*Clpp*^flox/flox^ female mice and Zp3-Cre male mice were generated previously using the C57BL/6 strain [16,26,27]. Female *Clpp*^flox/flox^ mice were mated with Zp3-Cre male mice to obtain *Clpp*^flox^/Zp3-Cre male mice. Female *Clpp*^flox/flox^ mice were mated with *Clpp*^flox^/Zp3-Cre male mice to obtain *Clpp*^flox/flox^/Zp3-Cre mice. Colony numbers were gradually expanded as needed. For simplicity, mice with oocyte-specific *Clpp* deletion, i.e., oocytes from *Clpp*^flox^/Zp3-Cre mice, will be referred to as *Clpp^−/−^* hereafter. Female *Clpp^−/−^* mice and WT mice were used for the experiments.

### 4.2. Assessment of Fertility

To assess the fertility of *Clpp*^−/−^ female mice, sexually mature female mice both from WT and of *Clpp*^−/−^ groups (*n* = 5 for 2-month-old and *n* = 4 for 6-month-old mice) were mated with male mice (12 weeks old) of proven fertility for 12 weeks at a 1:1 (female/male) ratio. To record the number of litters and pups, fertility test cages were monitored continuously.

### 4.3. Histomorphometric Analysis of Folliculogenesis in Ovaries

Ovaries from *Clpp*^−/−^ and WT female mice were stimulated with 10 IU of pregnant mare serum gonadotropin (PMSG) to obtain hematoxylin and eosin-stained sections for the assessment of folliculogenesis. Forty-eight hours after the injection, ovaries were dissected and fixed in 4% paraformaldehyde (Sigma, St. Louis, MO, USA) at 4 °C overnight and transferred into 70% ethanol for further processing. Dehydration, embedding, and sectioning (5 µm) steps were performed, respectively, and sections were stained with H&E. For each ovary, follicles containing oocytes with a visible nucleus on every fifth section were counted.

Follicles were categorized as primordial, primary, secondary, early antral, or antral follicles. Primordial follicles were defined as having a single layer of flat and squamous granulosa cells surrounding the oocyte. Primary follicles were described as surrounded by a single layer of cuboidal granulosa cells, whereas those surrounded by at least two layers of cuboidal granulosa cells, without a clear antrum, were considered to be secondary follicles. Follicles showing evidence of an antral cavity and four or more layers of surrounding granulosa cells were defined as early antral. Antral follicles were described as follicles containing an obviously defined, single, antral space.

### 4.4. Oocyte Collection

Intraperitoneal injection of 10 IU of PMSG (Sigma, St. Louis, MO, USA) was performed to stimulate oocyte development, and 44–48 h after the injection, ovaries of 2-month- and 9-month-old *Clpp*^−/−^ and WT female mice were used to obtain germinal vesicle (GV) oocytes. Retrieval was performed by puncturing with a 26-gauge needle in M2 medium (Sigma, St. Louis, MO, USA) supplemented by 10 µM milrinone (Sigma, St. Louis, MO, USA) to prevent meiotic resumption. The oocytes were collected by puncturing the whole ovary, and cumulus–oocyte complexes (COCs) were collected under a stereomicroscope. Oocytes were stripped to remove surrounding cumulus cells, and oocytes with a germinal vesicle (GV) were counted.

To collect metaphase 2 stage mature oocytes (MII), 10 IU of human chorionic gonadotropin (hCG; Sigma, St. Louis, MO, USA) was injected 48 h after the PMSG (Sigma, St. Louis, MO, USA) injection. Unfertilized MII oocytes were collected from oviducts as previously described 14–16 h after the hCG injection [46].

### 4.5. Quantification of mtDNA Copy Number in Oocytes

To quantify the mtDNA copy number in GV oocytes, a *Cox3* fragment was amplified and cloned into a pCR^TM^ 2.1-TOPO-colony vector (Invitrogen, Carlsbad, CA, USA) as previously described [47]. One-Shot TOP10 Chemically Component *E. coli* was transformed and grown overnight at 37 °C. A Qiagen Plasmid Isolation Kit (Cat No: 27104) was used for purification. DNA sequence analysis was conducted to confirm the insertion of the mtDNA fragment. The amount of plasmid DNA was quantified using a NanoDrop 2000 Spectrophotometer (Thermo Scientific, Waltham, MA, USA). Serial 10-fold dilutions were then performed to reach concentrations of 10^9^ to 10^2^ plasmid molecules per 3.3 µL, which were then used to generate a standard curve for the quantitative analysis.

Individual oocytes from WT and *Clpp*^−/−^ mice were lysed in 10 µL of lysis solution containing 125 µg/mL Proteinase K and 17 µM SDS in sterile water by incubating at 55 °C for 2 h. Inactivation of the lysis solution was performed for 10 min at 95 °C, and the mixture was used directly for downstream PCR as three replicates. Each 10 µL reaction consisted of 5 µL of SYBR Green Supermix (Bio-Rad Laboratories, Hercules, CA, USA), 0.3 µM of each primer, and one-third of oocytes’ total mtDNA. Each individual oocyte’s mtDNA copy number was extrapolated from the standard curve.

### 4.6. Quantification of ATP

To determine the ATP content of individual single GV oocytes, an ATP Bioluminescent Somatic Cell Assay Kit (Sigma, St. Louis, MO, USA) was used. Single GV oocytes were collected and mixed with 100 µL of somatic cell ATP Releasing Reagent, and samples were stored at −80 °C until processed. A total of 100 µL of ATP Assay Mix Working Solution was added individually to 96-well plate wells and stored at room temperature for 3–5 min. A total of 100 µL of fresh ATP Releasing Reagent was added to vials containing samples and swirled briskly. A total of 100 µL of this mixture was distributed individually to 96-well plates containing ATP Assay Mix Working Solution as two technical replicates. Dynex MLX Microliter Plate Luminometer (Dynex Technologies, Chantilly, VA, USA) was used to measure the amount of light emitted. ATP levels from an individual oocyte were calculated by comparison to a standard curve generated over the range of 2.5–500 fmol/100 µL.

### 4.7. Quantitative Reverse Transcription Polymerase Chain Reaction (qRT-PCR) 

Extraction of total RNA from collected GV oocytes was conducted using an RNAqueous Microkit (Thermo Fisher Scientific, Waltham, MA, USA). Reverse transcription was performed using a RETROscript Kit (Thermo Fisher Scientific, Waltham, MA, USA) according to the manufacturer’s protocol. PCR was performed as previously described [48]. Briefly, for each well, the mix was prepared with 5 µL of SYBR Green Supermix (Bio-Rad Laboratories), 3 µL of H_2_O, 0.5 µL of each primer, and 1 µL of cDNA. Initial denaturation was performed at 95 °C for 3 min followed by amplification at 60 °C for 30 s and final extension at 72 °C for 5 min, as per the protocol. Normalization was performed according to *β-actin* levels, and the 2^−ΔΔCT^ (cycle threshold) method was used to calculate the relative expression levels. Each experiment was repeated at least three times using individual animals from each genotype. Primers used for PCR reactions are provided in Table S1.

### 4.8. Statistical Analysis

Quantitative data were expressed as mean ± SEM. To assess for a significant difference between the two groups, Student’s *t*-test was performed. At least three independent biological replicates were used for each experiment unless otherwise specified. All statistical analyses were conducted using GraphPad Prism Software version 7.

## Figures and Tables

**Table 1:** Assessment of fertility in oocyte-specific *Clpp^−/−^* mice. Fertility of 2-month-old and 6-month-old female oocyte-specific *Clpp*^−/−^ (KO) and wild type (WT) mice was assessed by mating with WT males of proven fertility (male/female; 1:1) for 12 weeks. KO: *Clpp*^−/−^; * *p* < 0.05.

Assessment of Fertility in Oocyte Specific *Clpp^−/−^* Mice							
Genotype		n	Litters	Pups	Pups per Litter	Litter per Female	Pups per Female
2–month-old	WT	5	16	72	4.5 ± 0.11	3.2 ± 0.16	14.4 ± 3.76
	KO	5	10	36	3.6 ± 0.79	2 ± 0.54	7.2 ± 1.68 *
6–month–old	WT	4	12	69	5.75 ± 1.18	3 ± 0	17.25 ± 3.49
	KO	4	5	24	4.8 ± 0.47	1.25 ± 1.19 *	6 ± 2.44 *

## Data Availability

Data is contained within the article and Supplementary Materials.

## Supplementary Materials

The following supporting information can be downloaded at https://www.mdpi.com/article/10.3390/ijms25031866/s1.

## References

[1] Van Blerkom J. (2011). Mitochondrial function in the human oocyte and embryo and their role in developmental competence. Mitochondrion.

[2] Fragouli E., Wells D. (2015). Mitochondrial DNA Assessment to Determine Oocyte and Embryo Viability. Semin. Reprod. Med..

[3] BLledo J.A., Morales E., Garcia-Hernandez J., Ten A., Bernabeu J., Llacer R. (2018). Bernabeu, Comprehensive mitochondrial DNA analysis and IVF outcome. Hum. Reprod. Open.

[4] Reynier P., May-Panloup P., Chrétien M.-F., Morgan C.J., Jean M., Savagner F., Barrière P., Malthièry Y. (2001). Mitochondrial DNA content affects the fertilizability of human oocytes. Mol. Hum. Reprod..

[5] Van Blerkom J., Runner M.N. (1984). Mitochondrial reorganization during resumption of arrested meiosis in the mouse oocyte. Am. J. Anat..

[6] Nagai S., Mabuchi T., Hirata S., Shoda T., Kasai T., Yokota S., Shitara H., Yonekawa H., Hoshi K. (2006). Correlation of Abnormal Mitochondrial Distribution in Mouse Oocytes with Reduced Developmental Competence. Tohoku J. Exp. Med..

[7] Benedetti C., Haynes C.M., Yang Y., Harding H.P., Ron D. (2006). Ubiquitin-like protein 5 positively regulates chaperone gene expression in the mitochondrial unfolded protein response. Genetics.

[8] Zhao Q., Wang J., Levichkin I.V., Stasinopoulos S., Ryan M.T., Hoogenraad N.J. (2002). A mitochondrial specific stress response in mammalian cells. Embo J..

[9] Nargund A.M., Fiorese C.J., Pellegrino M.W., Deng P., Haynes C.M. (2015). Mitochondrial and Nuclear Accumulation of the Transcription Factor ATFS-1 Promotes OXPHOS Recovery during the UPRmt. Mol. Cell.

[10] Haynes C.M., Petrova K., Benedetti C., Yang Y., Ron D. (2007). ClpP Mediates Activation of a Mitochondrial Unfolded Protein Response in C. elegans. Dev. Cell.

[11] Brodie E.J., Zhan H., Saiyed T., Truscott K.N., Dougan D.A. (2018). Perrault syndrome type 3 caused by diverse molecular defects in CLPP. Sci. Rep..

[12] Key J., Gispert S., Koornneef L., Sleddens-Linkels E., Kohli A., Torres-Odio S., Koepf G., Amr S., Reichlmeir M., Harter P.N. (2022). CLPP Depletion Causes Diplotene Arrest; Underlying Testis Mitochondrial Dysfunction Occurs with Accumulation of Perrault Proteins ERAL1, PEO1, and HARS2. Cells.

[13] Fiumara A., Sorge G., Toscano A., Parano E., Pavone L., Opitz J.M. (2004). Perrault syndrome: Evidence for progressive nervous system involvement. Am. J. Med. Genet. A.

[14] Wang T., Babayev E., Jiang Z., Li G., Zhang M., Esencan E., Horvath T., Seli E. (2018). Mitochondrial unfolded protein response gene Clpp is required to maintain ovarian follicular reserve during aging, for oocyte competence, and development of pre-implantation embryos. Aging Cell.

[15] Gispert S., Parganlija D., Klinkenberg M., Dröse S., Wittig I., Mittelbronn M., Grzmil P., Koob S., Hamann A., Walter M. (2013). Loss of mitochondrial peptidase Clpp leads to infertility, hearing loss plus growth retardation via accumulation of CLPX, mtDNA and inflammatory factors. Hum. Mol. Genet..

[16] Esencan E., Cozzolino M., Imamoglu G., Seli E. (2020). Mitochondrial Stress Response Gene Clpp Is Not Required for Granulosa Cell Function. Antioxidants.

[17] Li G., Gu J., Zhou X., Wu T., Li X., Hua R., Hai Z., Xiao Y., Su J., Yeung W.S.B. (2023). Mitochondrial stress response gene Clpp deficiency impairs oocyte competence and deteriorate cyclophosphamide-induced ovarian damage in young mice. Front. Endocrinol..

[18] de Vries W.N., Binns L.T., Fancher K.S., Dean J., Moore R., Kemler R., Knowles B.B. (2000). Expression of Cre recombinase in mouse oocytes: A means to study maternal effect genes. Genesis.

[19] Ren Y., Suzuki H., Jagarlamudi K., Golnoski K., McGuire M., Lopes R., Pachnis V., Rajkovic A. (2015). Lhx8 regulates primordial follicle activation and postnatal folliculogenesis. BMC Biol..

[20] Martinelli P., Rugarli E.I. (2010). Emerging roles of mitochondrial proteases in neurodegeneration. Biochim. Biophys. Acta (BBA)-Bioenerg..

[21] Sheng X., Liu C., Yan G., Li G., Liu J., Yang Y., Li S., Li Z., Zhou J., Zhen X. (2022). The mitochondrial protease LONP1 maintains oocyte development and survival by suppressing nuclear translocation of AIFM1 in mammals. eBioMedicine.

[22] Lee Y.G., Kim H.W., Nam Y., Shin K.J., Lee Y.J., Park D.H., Rhee H.W., Seo J.K., Chae Y.C. (2021). LONP1 and ClpP cooperatively regulate mitochondrial proteostasis for cancer cell survival. Oncogenesis.

[23] Rankin T., Soyal S., Dean J. (2000). The mouse zona pellucida: Folliculogenesis, fertility and pre-implantation development. Mol. Cell. Endocrinol..

[24] de Figueiredo J.R., de Lima L.F., Silva J.R.V., Santos R.R. (2018). Control of growth and development of preantral follicle: Insights from in vitro culture. Anim. Reprod..

[25] Kasapoglu I., Seli E. (2020). Mitochondrial Dysfunction and Ovarian Aging. Endocrinology.

[26] Zhang M., Bener M.B., Jiang Z., Wang T., Esencan E., Scott Iii R., Horvath T., Seli E. (2019). Mitofusin 1 is required for female fertility and to maintain ovarian follicular reserve. Cell Death Dis..

[27] Zhang M., Bener M.B., Jiang Z., Wang T., Esencan E., Scott R., Horvath T., Seli E. (2019). Mitofusin 2 plays a role in oocyte and follicle development, and is required to maintain ovarian follicular reserve during reproductive aging. Aging.

[28] Ben-Meir A., Burstein E., Borrego-Alvarez A., Chong J., Wong E., Yavorska T., Naranian T., Chi M., Wang Y., Bentov Y. (2015). Coenzyme Q10 restores oocyte mitochondrial function and fertility during reproductive aging. Aging Cell.

[29] Chiang J.L., Shukla P., Pagidas K., Ahmed N.S., Karri S., Gunn D.D., Hurd W.W., Singh K.K. (2020). Mitochondria in Ovarian Aging and Reproductive Longevity. Ageing Res. Rev..

[30] Benkhalifa M., Ferreira Y.J., Chahine H., Louanjli N., Miron P., Merviel P., Copin H. (2014). Mitochondria: Participation to infertility as source of energy and cause of senescence. Int. J. Biochem. Cell Biol..

[31] Pang W., Zhang Y., Zhao N., Darwiche S.S., Fu X., Xiang W. (2013). Low expression of Mfn2 is associated with mitochondrial damage and apoptosis in the placental villi of early unexplained miscarriage. Placenta.

[32] Zhao J., Li Y. (2012). Adenosine triphosphate content in human unfertilized oocytes, undivided zygotes and embryos unsuitable for transfer or cryopreservation. J. Int. Med. Res..

[33] Purandhar K., Jena P.K., Prajapati B., Rajput P., Seshadri S. (2014). Understanding the role of heat shock protein isoforms in male fertility, aging and apoptosis. World J. Men’s Health.

[34] Jee B., Dhar R., Singh S., Karmakar S. (2021). Heat Shock Proteins and Their Role in Pregnancy: Redefining the Function of “Old Rum in a New Bottle”. Front. Cell Dev. Biol..

[35] Esfandiari N., Falcone T., Agarwal A., Goldberg J.M., Nelson D.R., Sharma R.K. (2002). Are heat shock proteins acting as modulators of pre-implantation mouse embryo development and apoptosis?. Fertil. Steril..

[36] Salmeri N., Viganò P., Cavoretto P., Marci R., Candiani M. (2023). The kisspeptin system in and beyond reproduction: Exploring intricate pathways and potential links between endometriosis and polycystic ovary syndrome. Rev. Endocr. Metab. Disord..

[37] Cejudo Roman A., Pinto F.M., Dorta I., Almeida T.A., Hernández M., Illanes M., Tena-Sempere M., Candenas L. (2012). Analysis of the expression of neurokinin B, kisspeptin, and their cognate receptors NK3R and KISS1R in the human female genital tract. Fertil. Steril..

[38] León S., Fernandois D., Sull A., Sull J., Calder M., Hayashi K., Bhattacharya M., Power S., Vilos G.A., Vilos A.G. (2016). Beyond the brain-Peripheral kisspeptin signaling is essential for promoting endometrial gland development and function. Sci. Rep..

[39] Mattam U., Talari N.K., Paripati A.K., Krishnamoorthy T., Sepuri N.B.V. (2021). Kisspeptin preserves mitochondrial function by inducing mitophagy and autophagy in aging rat brain hippocampus and human neuronal cell line. Biochim. Biophys. Acta Mol. Cell Res..

[40] Hu K.L., Chang H.M., Zhao H.C., Yu Y., Li R., Qiao J. (2019). Potential roles for the kisspeptin/kisspeptin receptor system in implantation and placentation. Hum. Reprod. Update.

[41] Evans M.C., Lord R.A., Anderson G.M. (2021). Multiple Leptin Signalling Pathways in the Control of Metabolism and Fertility: A Means to Different Ends?. Int. J. Mol. Sci..

[42] Zhang Y., Proenca R., Maffei M., Barone M., Leopold L., Friedman J.M. (1994). Positional cloning of the mouse obese gene and its human homologue. Nature.

[43] Mounzih K., Lu R., Chehab F.F. (1997). Leptin treatment rescues the sterility of genetically obese ob/ob males. Endocrinology.

[44] Kleinridders A., Lauritzen H.P., Ussar S., Christensen J.H., Mori M.A., Bross P., Kahn C.R. (2013). Leptin regulation of Hsp60 impacts hypothalamic insulin signaling. J. Clin. Investig..

[45] Hauffe R., Rath M., Schell M., Ritter K., Kappert K., Deubel S., Ott C., Jähnert M., Jonas W., Schürmann A. (2021). HSP60 reduction protects against diet-induced obesity by modulating energy metabolism in adipose tissue. Mol. Metab..

[46] Seli E., Lalioti M.D., Flaherty S.M., Sakkas D., Terzi N., Steitz J.A. (2005). An embryonic poly(A)-binding protein (ePAB) is expressed in mouse oocytes and early preimplantation embryos. Proc. Natl. Acad. Sci. USA.

[47] (2016). Pascale May-Panloup, Lisa Boucret, Juan-Manuel Chao de la Barca, Valérie Desquiret-Dumas, Véronique Ferré-L’Hotellier, Catherine Morinière, Philippe Descamps, Vincent Procaccio, Pascal Reynier, Ovarian ageing: The role of mitochondria in oocytes and follicles. Hum. Reprod. Update.

[48] Cozzolino M., Ergun Y., Seli E. (2022). Targeted Deletion of Mitofusin 1 and Mitofusin 2 Causes Female Infertility and Loss of Follicular Reserve. Reprod. Sci..

