# Supplementary material for: Mitochondrial Unfolded Protein Response Gene Clpp Is Required for Oocyte Function and Female Fertility
Source: Int J Mol Sci. 2024 Feb 3;25(3):1866. doi: 10.3390/ijms25031866 (PMC10855406; doi:10.3390/ijms25031866)
Supplement: Supplementary file 1 [file ijms-25-01866-s001.zip › Table S1. Primers_OKO.pdf]

Table S1. The list of primers used for quantitative RT-PCR

Gene	TaqMan assay number or Primer sequences (5' to 3'; F, forward; R, reverse)
β-actin	Mm00607939_s1
β-actin	F: GGCTGTATTCCCCTCCATCG<br>R: CCAGTTGGTAACAATGCCATGT
Atp5a1	Mm00431960_m1
Cox1	Mm04225243_g1
Dnaja3	Mm00469723_m1
Hspd1	F: AGTGGATGACCTCGTGTTATGC<br>R: GGATCTAGTTTCTGGCCTCTTCG
Hspe1	F: TGGTAATCTTTAGCGGTGCTC<br>R: GGAGGACTTTATCCCACAGC
Ndufv1	Mm00504941_m1
Uqcrc2	Mm00445961_m1
Sdhb	Mm00458272_m1
Cox3	F: TTTGCAGGATTCTTCTGAGC<br>R: TGAGCTCATGTAATTGAAACACC
