# Supplementary figures and images for: Mitochondrial Unfolded Protein Response Gene Clpp Is Required for Oocyte Function and Female Fertility
Source: Int J Mol Sci. 2024 Feb 3;25(3):1866. doi: 10.3390/ijms25031866 (PMC10855406; doi:10.3390/ijms25031866)

Figure S2. mRNA expression levels of WT and *Clpp*<sup>-/-</sup> GV oocytes

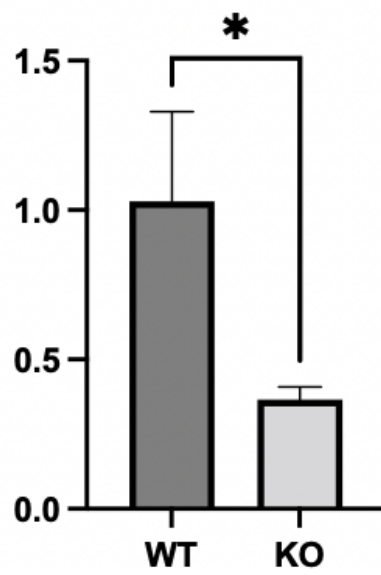

Supplement: Supplementary file 1 [file ijms-25-01866-s001.zip › Figure S1.pdf]
